# Polymyxin B and fusidic acid, a novel potent synergistic combination against *Klebsiella pneumoniae* and *Escherichia coli* isolates with polymyxin B resistance

**DOI:** 10.3389/fmicb.2023.1220683

**Published:** 2023-10-11

**Authors:** Shuying Chen, Peiyao Zhou, Chunyang Wu, Jie Wang, Ying Zhou, Jiao Zhang, Bingjie Wang, Huilin Zhao, Lulin Rao, Meilan Li, Fangyou Yu, Chunchan Lin

**Affiliations:** ^1^Department of Clinical Laboratory, Key Laboratory of Clinical Laboratory Diagnosis and Translational Research of Zhejiang Province, The First Affiliated Hospital of Wenzhou Medical University, Wenzhou, Zhejiang, China; ^2^Department of Respiratory Medicine, The First Affiliated Hospital of Wenzhou Medical University, Wenzhou, Zhejiang, China; ^3^Affiliated Hangzhou First People’s Hospital, Zhejiang University School of Medicine, Hangzhou, Zhejiang, China; ^4^Department of Clinical Laboratory Medicine, Shanghai Pulmonary Hospital, Tongji University School of Medicine, Shanghai, China; ^5^Department of Respiratory Medicine, Shanghai Pulmonary Hospital, Tongji University School of Medicine, Shanghai, China

**Keywords:** polymyxin B, fusidic acid, *Klebsiella pneumoniae*, resistance, synergistic effect

## Abstract

The increasing prevalence of multidrug-resistant (MDR) Gram-negative bacteria and comparatively limited options of antibiotics pose a major threat to public health worldwide. Polymyxin B is the last resort against extensively resistant Gram-negative bacterial infections. However, a large number of Gram-negative bacteria exhibited high-level resistance to Polymyxin B, bringing challenges for antimicrobial chemotherapy. Combination therapies using polymyxins and other antibiotics are recommended to treat multidrug-resistant pathogens. In this study, we selected Gram-negative bacterial strains, including *Klebsiella pneumoniae* and *Escherichia coli*, to explore whether fusidic acid and polymyxin B have a synergistic killing effect. Through broth microdilution, we observed that minimum inhibitory concentrations (MICs) against polymyxin B in the isolates tested were significantly reduced by the addition of fusidic acid. Notably, chequerboard analysis indicated a synergistic effect between polymyxin B and fusidic acid. In addition, subsequent time-kill experiments showed that the combination of polymyxin B and fusidic acid was more effective than a single drug in killing bacteria. Finally, our investigation utilizing the murine model revealed a higher survival rate in the combination therapy group compared to the monotherapy group. Our research findings provide evidence of the synergistic effect between polymyxin B and fusidic acid. Fusidic acid was shown to increase the sensitivity of multi-drug resistant *E. coli* and *K. pneumoniae* to polymyxin B, thereby enhancing its bactericidal activity. This study provides new insights into a potential strategy for overcoming polymyxin B resistance, however, further investigations are required to evaluate their feasibility in real clinical settings.

## Introduction

In recent years, the escalating prevalence of drug-resistant gram-negative bacteria has emerged as a major threat to global human health and public environmental problems worldwide (Lee et al., 2017). The threat of antibiotic resistance rendered patients at risk of ineffective treatment and increasing healthcare costs. Carbapenems, including imipenem, ertapenem, and meropenem, have been established as the preferred antibiotics for managing severe infections caused by multidrug-resistant (MDR) Gram-negative pathogens (Bergen et al., 2012; Zhanel et al., 2022). However, irrational utilization of carbapenems has facilitated the emergence of carbapenem-resistant Enterobacteriaceae (CRE), compounding clinical treatment challenges (Zhang et al., 2018; Suay-Garcia and Perez-Gracia, 2019; Wang et al., 2020). Thus, novel antibiotics are urgently needed to combat this challenge.

Polymyxins are generally regarded as the final recourse for the treatment of infections that are induced by CRE (Candan and Aksoz, 2015; Aguayo et al., 2016). Although the precise mechanism of antibacterial action of polymyxins remains unclear, it is widely acknowledged that polymyxin B and colistin (polymyxin E) are generally classified as cationic antimicrobial lipopeptides (CAMPs) that can effectively target a wide range of multidrug-resistant bacteria by disrupting the outer membrane (OM) barrier through lipid A binding (Couet et al., 2012; Gregoire et al., 2017). Although earlier studies suggested that polymyxin B exhibited a low incidence of bacterial resistance, recent evidence points to a marked increase in resistance rates, posing significant challenges to antibiotic treatment options (Cai et al., 2012; Karaiskos et al., 2017). The appearance of plasmid-borne resistant gene *mcr*-1 and mutations in chromosomal genes, phoPQ and pmrAB, have been implicated in the mechanism of resistance to polymyxin B (Li et al., 2020; Mmatli et al., 2022). Although the resistance rate of polymyxin B has been increasing annually, the combination therapy of polymyxin B with other drugs remains an effective strategy for the treatment of CRE infections (Rigatto et al., 2019; Fedrigo et al., 2021).

Fusidic acid, a natural steroid antibiotic, was initially isolated from the fungus Fusidium coccineum in the early 1960s, exhibiting potent activity against gram-positive bacteria (Fernandes and Pereira, 2011). As is well known, fusidic acid is a well-known inhibitor of elongation factor G (EF-G) function. Fusidic acid can impede bacterial growth through its inhibitory actions (Fernandes, 2016). Gram-negative bacteria are naturally resistant to fusidic acid. It was reported that the use of fusidic acid in combination with other drugs has the potential to be a new treatment strategy (Grohs et al., 2003).

In this study, we aimed to evaluate the potential synergistic effect of combining polymyxin B and fusidic acid against strains of *Escherichia coli* and *Klebsiella pneumoniae* that exhibit resistance to polymyxin B.

## Materials and methods

### Strains of bacteria and reagents

The strains were isolated from animals and clinical samples, including 15 *E. coli* and 26 *K. pneumoniae* isolates analyzed by MALDI-TOF mass spectrometry. These isolates were retrospectively retrieved from several tertiary hospitals in China and agricultural culture collections from July 2015 to December 2021. Polymyxin B and fusidic acid were purchased from Solarbio (Beijing, China).

### MIC assays

The Minimum Inhibitory Concentration (MIC) of Polymyxin B and fusidic acid were determined using broth microdilution in 96-well microtiter plates with freshly prepared Mueller-Hinton broth (Solarbio, Beijing, China). The concentration range of fusidic acid was from 1 μg/mL to 1024 μg/mL, the concentration range of polymyxin B was from 0.25 μg/mL to 64 μg/mL. The bacterial samples were inoculated at a concentration of 5 × 10^5^ colony-forming units (CFU) per milliliter, and a total volume of 200 μL was used. The microtiter plates were then incubated at 37°C for 20 h. *E. coli* ATCC 25922 was used as a quality control strain. The breakpoint for Polymyxin B was ≤2 μg/mL according to EuropeanCommittee on Antimicrobial Susceptibility Testing (EUCAST) (EUCAST, 2020), while the Clinical and Laboratory Standards Institute (CLSI) guidelines (CLSI, 2020) for fusidic acid in *E. coli* and *K. pneumoniae* have not yet been established due to inherent resistance in Gram-negative bacteria.

### Chequerboard assays

The chequerboard broth microdilution method was performed to study the interaction between the polymyxin B and fusidic acid using 96-well plates. Fusidic acid was serially diluted 1:2 dilutions in horizontal direction (twelve dilutions in total), while Polymyxin B serially diluted 1:2 dilutions toward the vertical direction (eight dilutions in total). Then, serial dilutions were loaded into 96-well plates to obtain combinations of two compounds with different concentrations, with the addition of 100 μL of the bacterial solution (making an ultimate inoculum of 5 × 10^5^ CFU/mL). The fractional inhibitory concentration (FIC) index was determined according to the equation: FIC of drug A = MIC of drug A in combination/MIC of drug A alone, FIC of drug B = MIC of drug B in combination/MIC of drug B alone, and FIC index = FIC of drug A + FIC of drug B. The FIC index values were interpreted as follows: antagonism = FIC index >4.0, no interaction = FIC index >0.5–4.0 and synergistic effects = FIC index ≤0.5.

### Time-kill assays

Time-kill analyses were carried out according to CLSI guidelines. Specifically, *K. pneumoniae* or *E. coli* overnight cultures were diluted 50–100 times in 20 mL of MHB and incubated for 3–4 h until reaching a density of 0.55 McFarland units. The cultures were then transferred to sterile borosilicate glass tubes and treated with either Polymyxin B, fusidic acid, or a combination of both. An equal amount of the sample was taken and diluted appropriately by a certain factor at predetermined time points (0, 0.5, 1, 2, 4, 6, and 24 h). Subsequently, 100 μL diluted sample was spread onto an MHA plate and incubated at 37°C for 24 h. Synergy was defined as a reduction of ≥2log10 in bacterial growth observed in combination treatment compared to the most effective monotherapy.

### Murine infection model

BALB/C mice (6 weeks old, female) were randomly divided into three groups (*n* = 6 per group), monotherapy group, combination therapy group and control group. FK3009 isolate was cultured overnight in Luria-Bertani (LB) broth. The overnight cultures were then diluted 200-fold and reinoculated into LB media, where they were grown to logarithmic phase. The isolate was subsequently washed three times with normal saline (NS) solution. Then, mice were infected via tail vein injection with 100 μL NS containing 1.0 × 10^8^ bacterial cells suspended. Mice injected with 100 μL sterile NS solution were used as control. After a 2-h period, monotherapy group and combination therapy group were injected polymyxin B (1.28 mg/kg) alone or both polymyxin B and fusidic acid (2.56 mg/kg). The mice were then observed at hourly intervals after the infection.

### Detection of resistance genes

The polymerase chain reaction (PCR) was used to amplify the resistance genes and the PCR product was sent for sequencing. Primers used in the experiment were *mcr-1*-F (5′-ATCAGCCAAACCTATCCC-3′) and *mcr-1*-R (5′-ACGCCACCACAGGCAGTA-3′).

## Results

### Bacterial isolates

We selected bacterial strains from hospitals and farms, and identified experimental strains through antimicrobial susceptibility testing. Our investigation included 26 isolates of *K. pneumoniae* and 15 isolates of *E. coli*, originating from both human and animal strains. Among the *K. pneumoniae* isolates, 50% displayed resistance to polymyxin B, while only 33.4% of *E. coli* isolates demonstrated resistance to polymyxin B (Table 1). Our PCR validation revealed that the *mcr*-1 encoding resistance to polymyxin B was detected in isolates of *E. coli* from 4 out of 15 cases (26.7%), whereas only one isolate of *K. pneumoniae* tested positive for the gene.

**Table 1:** Strain information and minimum inhibitory concentrations (MIC) of polymyxin B and fusidic acid against bacterial isolates in this study.

Isolate	Source	MIC					Polymyxin susceptibility and mechanism of resistance
		FA	PB	PB in the presence of 32 μg/mL FA	PB in the presence of 64 μg/mL FA	PB in the presence of 128 μg/mL FA	
*Klebsiella pneumoniae*							
LYM	Human	512	>64	2	2	1	Uncharacterized
LQP	Human	1024	64	2	2	1	Uncharacterized
GBC	Human	128	2	1	1	0.5	Susceptible
XGE	Human	32	2	0.5	0.5	0.5	Susceptible
N816	Human	>1024	>64	32	4	4	*mcr-1*
1530	Human	>1024	>64	2	2	1	Uncharacterized
1570	Human	128	4	1	1	0.5	Uncharacterized
1582	Human	>1024	>64	4	2	2	Uncharacterized
1584	Human	>1024	>64	4	4	2	Uncharacterized
1625	Human	1024	4	1	1	1	Uncharacterized
1769	Human	512	>64	1	1	1	Uncharacterized
FK3009	Human	512	>64	1	1	1	Uncharacterized
FK3035	Human	>1024	8	1	1	1	Uncharacterized
FK3062	Human	>1024	32	2	2	2	Uncharacterized
FK3064	Human	>1024	16	1	1	1	Uncharacterized
FK3101	Human	512	2	1	0.5	0.5	Susceptible
FK3061	Human	1024	2	1	1	0.5	Susceptible
FK3021	Human	512	2	1	1	0.5	Susceptible
FK3109	Human	512	2	0.5	0.5	0.5	Susceptible
FK3143	Human	>1024	2	1	0.5	0.5	Susceptible
FK3048	Human	512	2	1	1	0.5	Susceptible
1521	Human	512	2	0.5	0.5	0.5	Susceptible
N1060	Human	>1024	2	1	0.5	1	Susceptible
N1071	Animal	>1024	2	2	0.5	0.5	Susceptible
N597	Human	>1024	2	2	1	0.5	Susceptible
N656	Human	512	2	1	1	0.5	Susceptible
*Escherichia coli*							
N6	Animal	>1024	4	1	1	1	*mcr-1*
N12	Animal	512	64	2	2	1	Uncharacterized
N16	Animal	512	>64	4	2	2	*mcr-1*
N19	Animal	>1024	>64	2	2	2	*mcr-1*
N21	Animal	256	64	2	1	1	*mcr-1*
1120	Human	512	2	2	1	0.5	Susceptible
1124	Human	>1024	1	2	1	0.5	Susceptible
1143	Human	>1024	1	2	1	1	Susceptible
1144	Human	>1024	1	1	1	1	Susceptible
1147	Human	512	1	2	0.5	1	Susceptible
1188	Human	512	1	1	1	0.5	Susceptible
1324	Human	>1024	2	2	1	1	Susceptible
1117	Human	>1024	1	1	0.5	0.5	Susceptible
1118	Human	1024	1	1	0.5	0.5	Susceptible
1119	Human	>1024	1	1	1	1	Susceptible

PB Stands for polymyxin B and FA stands for fusidic acid.

### Fusidic acid is a sensitizer for polymyxin B

Table 1 presented the MIC values of 41 bacterial strains toward polymyxin B and fusidic acid. The MICs of fusidic acid for all other strains were ≥ 128 μg/mL. In most strains of *K. pneumoniae*, we observed a decrease in the MICs of polymyxin B due to the addition of 32 μg/mL of fusidic acid. Notably, for some highly resistant isolates, whose MICs of fusidic acid >64 μg/mL, the MIC values of the polymyxin B in the presence of fusidic acid were reduced to 1 μg/mL or 2 μg/mL. The results indicated that the magnitude of the reduction in MIC values was positively correlated with the concentration of fusidic acid, as observed following the addition of either 64 or 128 μg/mL of fusidic acid. Additionally, the addition of fusidic acid was found to have a similar effect on the growth of *E. coli*, indicating that it may increase sensitivity to polymyxin B regardless of the strains’ origin or the underlying mechanism of polymyxin B resistance. These findings suggested that fusidic acid has the potential to enhance the susceptibility of strains to polymyxin B.

### Synergistic effect of polymyxin B and fusidic acid

Chequerboard assays were conducted to evaluate the potential synergism of the polymyxin B and fusidic acid, and FICI scores are shown in Table 2. Our findings showed varying levels of synergy among 12 *K. pneumoniae* isolates, with FICI scores ranging from 0.063 to 0.281. Conversely, three isolates of *K. pneumoniae* demonstrated no interaction, as shown by FICI scores ranging from 0.5313 to 0.7500. Among isolates of *E. coli*, our results indicated a possible synergy between polymyxin B and fusidic acid. Overall, our chequerboard assays suggested a potential synergistic interaction between the two drugs.

**Table 2:** FIC index values for polymyxin B and fusidic acid against MDR bacterial isolates.

Isolate	FIC of Polymyxin B	FIC of Fusidic acid	FIC index	Interpretation
LYM	<0.0625	0.0019	<0.0644	Synergistic
LQP	0.0625	0.0010	0.0635	Synergistic
N816	<0.0625	<0.0625	<0.125	Synergistic
1530	<0.0312	<0.0039	<0.0352	Synergistic
1570	0.5000	0.0312	0.5313	No interaction
1582	<0.0625	<0.0156	<0.0781	Synergistic
1584	<0.0625	<0.0312	0.0938	Synergistic
1625	0.2500	0.0009	0.2510	Synergistic
1769	<0.0625	0.0625	<0.1250	Synergistic
FK3009	<0.0156	0.0078	<0.0234	Synergistic
FK3035	0.1250	<0.0009	<0.1260	Synergistic
FK3062	0.0625	<0.0009	<0.0635	Synergistic
FK3064	0.0625	<0.0009	<0.0635	Synergistic
N6	0.2500	0.0312	0.2812	Synergistic
N12	0.2500	0.0019	0.2519	Synergistic
N16	<0.2500	0.0019	<0.2519	Synergistic
N19	<0.0312	<0.0312	<0.0625	Synergistic
N21	0.0625	0.0039	0.0664	Synergistic

### Time-kill results of polymyxin B and fusidic acid against *Klebsiella pneumoniae* and *Escherichia coli*

**Figure 1:**
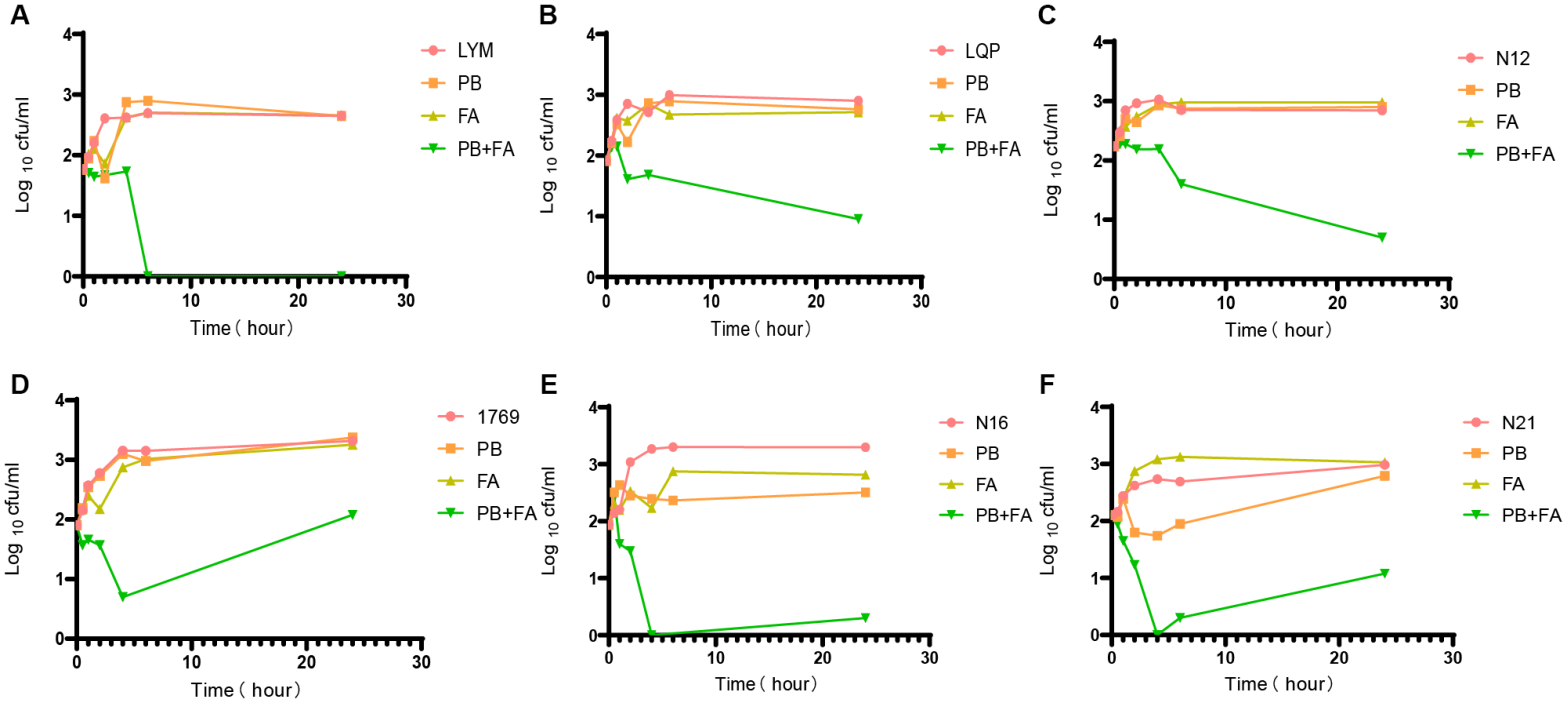
Time-kill experiments. Bacterial colony forming units in the absence of drug, and in the presence of 1/4 MIC polymyxin B, in the presence of 32 μg/mL fusidic acid and in the presence of both drugs after different periods of incubation PB = polymyxin B, FA = fusidic acid. Data presented are Log_10_CFU/mL mean values from the results of two independent experiments. Results for all 6 strains tested are presented. **(A)** LYM, **(B)** LQP, **(C)** N12, **(D)** 1769, **(E)** N16, and **(F)** N21.

We determined the optimal concentration of fusidic acid in combination with polymyxin B using chequerboard assays. Except for a few strains, the optimal concentration of polymyxin B for the remaining bacterial strains was determined to be 1/4 MIC, when the concentration ranged between 32 and 128 μg/mL. As a result, we selected the 1/4 MIC of polymyxin B and the concentration of fusidic acid at 32 μg/mL for conducting time-kill experiments. Whilst neither drug alone exhibited complete inhibition of bacterial growth, the combined treatment of polymyxin B and fusidic acid demonstrated a remarkable reduction in the number of viable bacteria. This effect was particularly pronounced for strains LYM, LQP, 1769, N12, N16 and N21, at 1 h, 2 h, 4 h, 6 h, and 24 h (Figure 1). Despite the presence of subtle distinctions, time-kill assays demonstrated the rapid bactericidal activity of the combination therapy comprising polymyxin B and fusidic acid.

**Figure 2:**
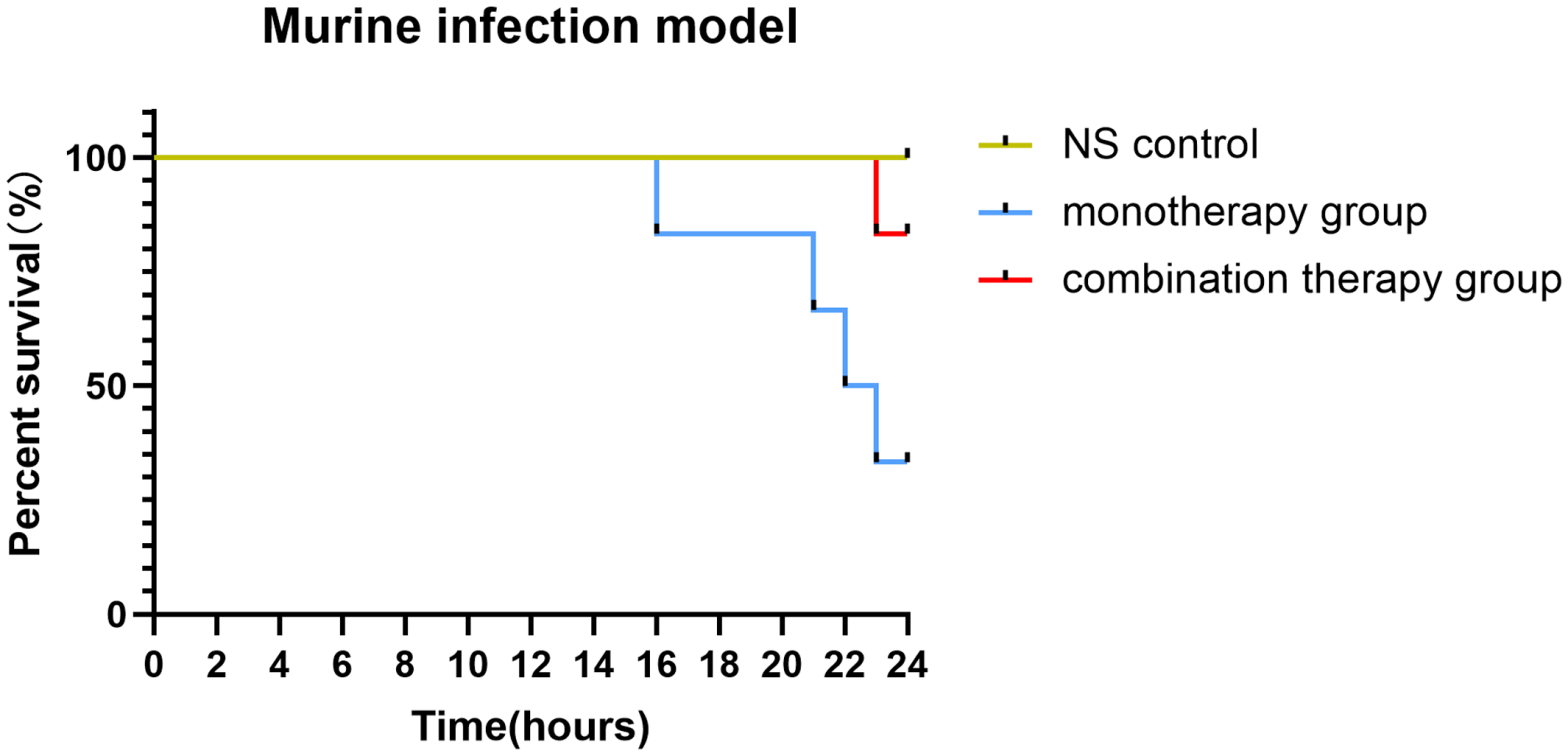
The survival rates of mice infected with FK3009 isolate.

### Murine infection model

The combination therapy of polymyxin and fusidic acid exhibited a synergistic effect *in vitro*, indicating that it is essential to verify the effects *in vivo*. Consequently, we conducted the murine infection model, employing the FK3009 isolate as the subject. FK3009 has shown high resistance to polymyxin B, and the MIC value of polymyxin B decreased significantly in the presence of fusidic acid. Within 24 h, the group treated with a combination of polymyxin B and fusidic acid exhibited a significantly higher survival rate (83%) compared to the group administered with polymyxin B alone (33%) (Figure 2). These results indicated that the combination of polymyxin and fusidic acid exhibited a significantly synergistic antibacterial effect against *Klebsiella pneumoniae in vivo*.

## Discussion

Alongside the increasing utilization of carbapenem, there is a growing concern about the development of novel resistance to antibiotics used against Gram-negative bacteria. It is imperative to identify new antimicrobials that can effectively combat against CRE. Polymyxin B has been considered as the last-resort treatment option for MDR infections, including infections caused by CRE (Li et al., 2006; Lim et al., 2010). However, the reported epidemiological information suggests that many cases of polymyxin B-resistant strains are emerging worldwide at an alarming rate (Nation and Li, 2009; Moffatt et al., 2019).

Currently, the mechanisms for Polymyxin B resistance have not been clearly elucidated. The previous studies showed the following four principal findings. Firstly, numerous bacteria develop resistance to polymyxins by decreasing the alteration of the head group of lipid A. This modification is originally enabled by electrostatic interactions and is likely mediated by genes located on both the chromosomal and plasmid (Sarkar et al., 2007; Nordmann and Poirel, 2016; Moffatt et al., 2019). Secondly, another mechanism is the induction of efflux pump systems and barriers, which involves increasing the production of capsular polysaccharides, mutations that alter the expression of efflux pumps, and the presence of modified porins that reduce outer membrane permeability, among others (Gregoire et al., 2017; Moffatt et al., 2019). In addition, enzymes produced by bacteria can facilitate the degradation of polymyxin B, leading to the reversal of the resistance phenotype associated with polymyxin B (Sarkar et al., 2007; Moffatt et al., 2019). Finally, the heterogeneity of resistance mechanisms also plays a crucial role in the development and spread of drug resistance (Rigatto et al., 2019).

Several studies have demonstrated that the combinations of polymyxin B and various traditional antibiotics result in increased antibacterial efficacy, highlighting the potential of combination therapy in addressing drug-resistant bacteria (Liu et al., 2020; Allend et al., 2022; Almutairi, 2022). Tian et al. (2021) observed the combination of polymyxin B and tigecycline reduced both of their MICs, indicating that tigecycline combined with polymyxin B may be a promising strategy. Phee et al. (2019) first identified colistin/fusidic acid as a novel strategy against Multidrug-resistant *Acinetobacter baumannii* (MDR-AB). The combination treatment remains effective even at low concentrations, which is clinically feasible while minimizing drug toxicity. The antibiotic fusidic acid targets EFG, thereby obstructing protein synthesis (Kinoshita et al., 1968). Moreover, fusidic acid exhibits an immunoregulatory effect primarily by impeding cytokine production, eradicating bacteria, and treating various inflammatory responses instigated by bacterial toxins (Kraus and Burnstead, 2011). Fusidic acid has been shown highly effective against staphylococcus, and is usually administrated via oral and parenteral routes (Mlynarczyk-Bonikowska et al., 2022). However, it is notable that gram-negative bacteria have an inherent resistance to fusidic acid.

According to the pharmacokinetics of fusidic acid, administration of a single 500 mg oral dose on an empty stomach results in a blood concentration of approximately 30 μg/mL within 2 to 3 h. For dosing regimens involving oral administration every 8 h over a span of 4 consecutive days, the blood concentration can reach 50–100 μg/mL. Given that large initial doses autoinhibit the clearance of fusidic acid, a steady state can be achieved earlier with dosing regimens that contain higher doses (Bulitta et al., 2013). Therefore, in this study, we tested MIC of polymyxin B against 41 isolates in the presence of 32 μg/mL, 64 μg/mL and 128 μg/mL fusidic acid in order to simulate the drug concentration achieved in human plasma and inhibit the clearance of fusidic acid. Recent pharmacodynamic (PD) and pharmacokinetic (PK) findings concerning polymyxin B indicate that the use of polymyxin B monotherapy is insufficient in achieving consistent and effective plasma concentrations (Garonzik et al., 2011). Notably, monotherapy may lead to resistance development, particularly when a concentration exceeds clinically feasible levels (Bergen et al., 2008). Low levels of resistance evolve repeatedly when low concentration of polymyxin B is applied, but this resistance is reversed after the antibiotic is removed. In contrast, super-MIC levels of polymyxin B (≥4 × MIC) drive the evolution of irreversible resistance. Therefore, combination therapy is recommended to enhance antimicrobial activity and counter resistance (Zhao et al., 2022). In our study, we compared the effectiveness of fusidic acid and polymyxin B in combination therapy and monotherapy for treating *E. coli* and *K. pneumoniae* infections. Additionally, a murine model was employed to further assess the efficacy of the combination therapy of polymyxin and fusidic acid.

In our study, *E. coli* with resistance to polymyxin B were isolated from animals, while *K. pneumoniae* with resistance to polymyxin B were isolated from humans. We also found that the combined use of polymyxin B and fusidic acid was more effective in bacterial killing than single-drug therapies. Notably, this synergistic effect was observed across various bacterial strains, as evidenced by the low FIC index of less than 0.5 in 5 *E. coli* and 12 *K. pneumoniae* strains tested in the chequerboard assay (Table 2). Besides, a higher survival rate was shown in the mice receiving combination therapy compared to those treated with polymyxin B alone.

Our findings suggested that the combination of fusidic acid and polymyxin B has a potential for broad clinical application value. Meanwhile, the drug resistance of polymyxin caused by the usage of polymyxin in animal husbandry deserves attention. Therefore, we urge a decrease in the administration of polymyxin B in animal husbandry.

## Data availability statement

The original contributions presented in the study are included in the article/supplementary material, further inquiries can be directed to the corresponding authors.

## Ethics statement

The animal study was approved by Laboratory Animal Ethics Committee of the First Affiliated Hospital of Wenzhou Medical University. The study was conducted in accordance with the local legislation and institutional requirements.

## Author contributions

SC, PZ, and CW: conceptualization, data curation, formal analysis, methodology, and writing–original draft. JW and YZ: data curation, methodology, and writing–original draft. JZ and BW: methodology. HZ: software. LR: formal analysis. FY and ML: conceptualization and writing–review and editing. CL: conceptualization, project administration, and writing–review and editing. All authors contributed to the article and approved the submitted version.
